# Damage Control Interventional Radiology in Liver Trauma: A Comprehensive Review

**DOI:** 10.3390/jpm14040365

**Published:** 2024-03-29

**Authors:** Fabio Corvino, Francesco Giurazza, Paolo Marra, Anna Maria Ierardi, Antonio Corvino, Antonio Basile, Massimo Galia, Agostino Inzerillo, Raffaella Niola

**Affiliations:** 1 Section of Radiology, Department of Biomedicine, Neuroscience and Advanced Diagnostics (BiND), University Hospital “Paolo Giaccone”, 90127 Palermo, Italy; massimo.galia@unipa.it (M.G.); agostino.inzerillo@unipa.it (A.I.); 2 Interventional Radiology Department, AORN “A. Cardarelli”, 80131 Naples, Italy; francesco.giurazza@aocardarelli.it (F.G.); raffaella.niola@aocardarelli.it (R.N.); 3 Department of Radiology, Papa Giovanni XXIII Hospital, 24127 Bergamo, Italy; pmarra@asst-pg23.it; 4 Department of Diagnostic and Interventional Radiology, Foundation IRCCS Cà Granda—Ospedale Maggiore Policlinico, 20122 Milan, Italy; annamaria.ierardi@policlinico.mi.it; 5 Medical, Movement and Wellbeing Sciences Department, University of Naples “Parthenope”, 80133 Naples, Italy; antonio.corvino@uniparthenope.it; 6 Radiology Unit 1, Department of Medical Surgical Sciences and Advanced Technologies “GF Ingrassia”, University Hospital “Policlinico-San Marco”, University of Catania, 95123 Catania, Italy; basile.antonello73@gmail.com

**Keywords:** liver trauma, damage control interventional radiology, transarterial embolization, hemodynamic instability

## Abstract

The liver is the second most common solid organ injured in blunt and penetrating abdominal trauma. Non-operative management (NOM) has become the standard of care for liver injuries in stable patients, where transarterial embolization (TAE) represents the main treatment, increasing success rates and avoiding invasive surgical procedures. In hemodynamically (HD) unstable patients, operative management (OM) is the standard of care. To date, there are no consensus guidelines about the endovascular treatment of patients with HD instability or in ones that responded to initial infusion therapy. A review of the literature was performed for published papers addressing the outcome of using TAE as the primary treatment for HD unstable/transient responder trauma liver patients with hemorrhagic vascular lesions, both as a single treatment and in combination with surgical treatment, focusing additionally on the different definitions used in the literature of unstable and transient responder patients. Our review demonstrated a good outcome in HD unstable/transient responder liver trauma patients treated with TAE but there still remains much debate about the definition of unstable and transient responder patients.

## 1. Introduction

Trauma is a global phenomenon; about 5 million people die worldwide each year due to an injury. Injuries also account for 17% of the disease burden in young adults aged between 15 and 60 years [1].

Interventional radiology (IR) plays a pivotal role in the management of trauma patients suffering from both blunt and penetrating injuries. In the last three decades, the focus on damage control resuscitation and damage control surgery has spared countless patients the morbidity of surgery, identifying which patients will benefit the most from a minimally invasive treatment strategy [2].

Prompt hemorrhage control with angioembolization (AE), as part of the damage control strategy, has been integrated into trauma resuscitation guidelines, as stated by the latest version of the Resources for Optimal Care of the Injured Patient from the American College of Surgeons Committee on Trauma [3].

The new concept of “damage control interventional radiology” (DCIR) proposed the availability of IR within less than 30 min in an emergency setting, trying to provide trauma patients with the best treatment as soon as possible to achieve the best possible outcome. In this context, even if according to the World Society of Emergency Surgery (WSES), hemodynamic (HD) unstable patients are an exclusive prerogative of surgical management, IR could be crucial in the management of HD unstable patients, especially for those who represent the transient responder class who need to receive endovascular hemostasis and subsequent resuscitation [4].

An earlier endovascular approach could also be possible due to the technological advancement achieved with the implementation of the trauma hybrid resuscitation suites that allow for the treatment of patients as soon as possible, both endovascular treatment alone or in combination with surgical procedures such as damage control surgery [5].

After the spleen, the most common solid organ injured in blunt and penetrating abdominal trauma is the liver; given its location and its relationship with other abdominal structures, the mortality rate is quite high at about 10–15% [6].

Non-operative management (NOM) is the current standard of care in the management of trauma liver patients who are HD stable and it consists of a basic “wait and see” attitude combined with blood replacement and systemic support. Imaging advancement has a major role in the success rate of NOM because it allows us to identify the injury grade, the presence of arterial hemorrhage and/or concomitant venous injury, which are crucial in the management algorithm [7]. To date, WSES classification in liver trauma patients with arterial hemorrhage or early pseudoaneurysm suggest AE as the primary treatment only in HD stable patients [8].

HD unstable and non-responder patients should undergo operative management (OM). Moreover, there is a “gray area” between stable and unstable patients, known as “transient responder patients”, in which NOM should be considered only in selected settings that provide the immediate availability of both surgeons and interventional radiologists, with continuous monitoring, ideally in an intensive care unit or emergency room setting [8].

The purpose of this systematic review is to determine the safety and efficacy of AE as the primary treatment for HD unstable or transient responder trauma liver patients with hemorrhagic vascular lesions, both as a single treatment and in combination with surgical treatment, focusing additionally on the different definitions of HD instability.

## 2. Materials and Methods

A literature review with a focus primarily on AE in the treatment of vascular lesions due to liver trauma, both blunt and penetrating trauma, in HD unstable patients was conducted from January 1980 to January 2024.

A systematic literature search was performed using PubMed, Google Scholar and the Cochrane Central Register of Controlled Trials databases for studies published on the role of IR in the management of liver trauma in HD unstable patients, both as a single treatment or in combination with surgical treatment. Medical subject headings (MeSH) and database-specific search terms for “liver trauma”, “liver embolization”, “hemodynamic status” and “damage control interventional radiology” were combined as follows: ((hepatic) OR (liver)) AND (trauma) AND (embolization) OR (embolisation) OR (angioembolization) AND (hemodynamic) AND (unstable); (((((((hepatic) OR (liver)) AND (trauma)) AND (embolization)) OR (embolisation)) OR (angioembolisation)) AND (hemodynamic)) AND (unstable); (((hepatic) OR (liver)) AND (trauma)) AND (damage control interventional radiology)

Supplemented articles were implemented by the ones obtained from the reference list of all relevant articles. We included only articles in the English language where it was possible to access to the full content.

**Figure 1:**
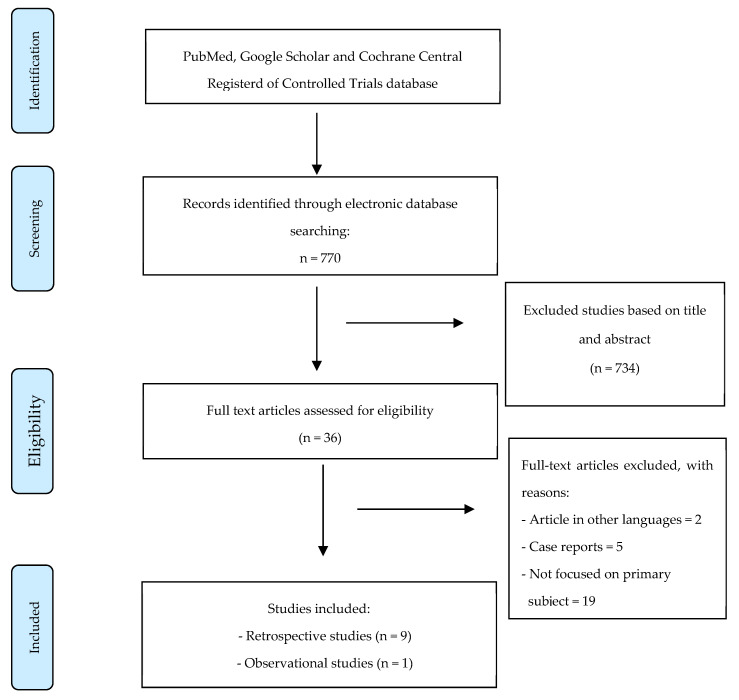
PRISMA flow diagram for systematic research and selection of studies included in the review.

Preferred Reporting Items for Systematic Reviews (PRISMA) (Figure 1) was used as the reference for data collection and the search was performed between December 2023 and January 2024, including all of the articles published until January 2024. Our review included all of the studies that evaluated the efficacy, safety and feasibility of endovascular treatment in HD unstable patients with liver trauma hemorrhage, focusing especially on the hemodynamic parameters from a more clinical perspective of the interventional radiology treatment.

The inclusion criteria in the selection of the studies were as follows:

- Hepatic bleeding from a traumatic cause, either blunt or penetrating;
- Endovascular treatment used alone or in combination with surgical procedures to treat only hepatic injuries;
- Description of the HD status of the patient, with a focus on articles where unstable patients were treated;
- Evaluation of the outcomes after embolization.

The following exclusion criteria were included:

- Case reports;
- Studies in which embolization for the treatment of liver bleeding was used in stable patients;
- Studies in which the population was pediatric;
- Studies where the endovascular treatment was used to treat non-traumatic liver injuries.

### 2.1. Outcomes

The primary outcomes evaluated included the clinical success rate, all-cause mortality and overall morbidity post-procedure among the HD unstable liver trauma patients treated with embolization. The common complications after endovascular treatment were considered, such as liver abscess/biloma formation, bile leak, gallbladder necrosis/acute cholecystitis, peritonitis and abdominal compartment syndrome. Moreover, the AAST classification was investigated to relate the presence of a correlation between the severity of the liver injury and the HD status of the patients.

### 2.2. Data Extraction

Two reviewers (F.C. and F.G.) screened titles first individually and then together to choose the appropriate articles. All of the data needed for the studies were extracted and tabulated after an in-depth reading process; the results are presented using descriptive statistics, and dichotomous and continuous variables are reported as absolute numbers, means, percentages, ranges and ratios as appropriate.

## 3. Results

According to the inclusion and exclusion criteria, initially, a total of 770 references were identified. The first evaluation of the title and abstract allowed us to exclude a total of 734 references. The remaining 36 articles were further considered for inclusion in the review and evaluated in the full-article review step. A total of 18 of them were excluded because they did not focus on the primary subject, 5 of them were excluded because they were case reports and 2 of them were excluded because they were not written in the English language. Finally, a total of 10 studies were included in the review [9,10,11,12,13,14,15,16,17,18]. Almost all of them were retrospective studies and only one was an observational study. Table 1 summarizes the main characteristics of the studies included and the number and demographic details of the enrolled patients.

### 3.1. Patient Demographics

We collected pooled data on 507 HD unstable liver trauma patients from 10 separate articles selected according to the inclusion and exclusion criteria; of these 507 patients, only 171 underwent a primary embolization treatment. Patients included in the studies had predominantly severe liver trauma. The American Association for the Surgery of Trauma (AAST) injury grade rate of the different studies is reported in Table 1; however, only six articles reported the AAST grade exact number of HD unstable trauma patients [19].

### 3.2. Hemodynamic Status

One of the main focus points of this review concerns the HD status of the patients and its definition; the different definitions available for the HD status of the patients among the 11 studies are summarized in Table 2. There were many differences between single studies in the definition of HD unstable/transient responder patients. Only one article identified HD unstable liver trauma patients using the Shock Index (SI), defined by the ratio of the heart rate (HR) to the systolic blood pressure (sBP) [9]. The majority of articles used the sBP but with different values. Aoki et al. defined unstable patients as those who had an sBP < 90 mmHg upon hospital arrival and received blood transfusion within the first 24 h after arrival [10]. In the same way, Otsuka et al. considered unstable patients those who displayed persistent hypotension with an sBP < 90 mmHg following primary resuscitation and Ogura et al. considered the cut-off of an sBP maintained at 70 mmHg. The first study considered stabilized patients as those who responded to resuscitative therapy and the second two, instead, considered HD unstable patients stabilized with resuscitative endovascular balloon occlusion of the aorta (REBOA) [13,14]. Inukai et al. and Mitsusada et al. considered HD unstable patients first as those that reached an sBP ≥ 90 mmHg for even a second after rapid fluid infusion or blood transfusion, and second as those with a value of sBP ≥ 80 mmHg after resuscitative therapy [12,13,14,15].

### 3.3. Mortality and Morbidity

All of the outcome measures were evaluated and are reported in Table 3. The number of patients with failure of the arterial embolization procedure were reported only in five studies, with a total number of four patients and a rate of 2,33%. Moreover, in only four studies, there was no report of mortality in HD liver trauma patients; a total average of 9.3% of mortality with a range between 3.2 and 36% was reported. The most frequent cause of mortality was not due to liver injury, but it was related to a concomitant severe head trauma with very low grade on the Glasgow Coma Scale (GCS) at admission.

Only in four studies was it not possible to determine the number of complications related to the embolization procedure (Table 4). The average complication rate was 34.5%, with a range between 15.7% and 80%. The most common complication reported was liver abscess/biloma with an incidence of 12.8% and a range between 5.26 and 36%. Bile leakage was reported with a mean incidence of 3% and a range between 3% and 37.5%. Gallbladder necrosis was reported in seven studies with a mean incidence of 5.3% and a range between 1% and 66%. Peritonitis was reported with a mean incidence of 6.4% and a range between 5.2 and 54%. Finally, abdominal compartment syndrome complication was reported in only six studies and occurred with a mean incidence of 1.16% and a range between 1.16% and 20%. Hepatic ischemia was not evaluated as a complication because it is a well-known outcome due to the embolization procedure. No study took into consideration the individualized complication rate according to the embolic agent used.

## 4. Discussion

WSES guidelines recommend TAE as the first-line therapy in HD stable patients with blunt or penetrating liver trauma; on the other hand, operative management (OM) (level of evidence I) is recommended in HD unstable patients with no indication for NOM [8]. To date, there are no comparative studies of TAE and OM in HD unstable liver trauma patients.

One of the main issues of this review is the definition of unstable patients, where our review found notable heterogeneity in the definition between the individual studies; the majority of the studies considered in our review define unstable patients as those who can benefit from TAE and those who initially respond to massive fluid and blood resuscitation, according to WSES guidelines, are categorized as transient responder patients. These patients could be stable enough to undergo a CT scan and can also be managed non-surgically. Trauma protocols of every hospital are based on the Advanced Trauma Life Support (ATLS) program, created by the American College of Surgeons Committee on Trauma. In the ATLS program, the definition of shock state is related to the evaluation of sBP, HR and base deficit (BD); however, the cut-off points of these vital signs have been disputed by some authors [20]. A recent systematic review points out that HD stability is the most important factor in the assessment of trauma patients; however, no consensus on the definition of HD stability between individual trauma centers has been demonstrated in the literature, pointing out that only a limited number of patients can be classified into the current ATLS shock classification [1]. However, further high-quality studies are needed to confirm this statement and a specific indication about the treatment of this kind of patient should be addressed more extensively by new guideline revisions.

A recent observational study demonstrated a >50% change in the management of HD unstable trauma patients subjected to a prior contrast-enhanced computed tomography (CT) scan. CT scans may have a role in detecting and managing such patients appropriately; however, in this paper, there is no clear definition of HD instability and whether the patients are partial responders or not to the initial resuscitative management [21].

Our review demonstrates a good outcome in partially responding unstable patients treated with TAE and NOM in institutions where there is 24 h availability of an IR team that could perform a prompt embolization treatment. Tamura et al. demonstrated a similar outcome in HD unstable liver trauma patients (who initially responded to infusion therapy) treated with TAE and NOM as compared to stable liver trauma patients treated with NOM. TAE for HD unstable patients with liver injury does not increase the mortality rates (6% in this series compared to 3–8% of an observational study) [22]. Moreover, the TAE group demonstrates fewer massive transfusions and shorter intensive care unit (ICU) stays than the OM group. However, in a multivariate analysis, the only predictor of ICU stay and massive transfusion was the initial HD status, and thus, they may not be related to treatment [9].

A recent systematic review and meta-analysis demonstrated a good clinical success rate (91%), bleeding resolution and the absence of further intervention, and a low mortality rate (7%) due to NOM in solid organ trauma HD unstable patients; moreover, one of the main inclusion criteria for TAE in HD unstable patients is an initial response to the initial resuscitative management that allows the target blood pressure to reached that is required to access the angiographic suite and perform the procedure [23]

One of the most important factors in determining the success rate of TAE in unstable patients depends on the time elapsed from CT diagnosis and the endovascular procedure. A cohort study reported that the presence of a pathway where CT study and TAE were performed within 30 min in unstable patients who were either complete or partial transient responders to the resuscitative protocol, as well as in patients who were in a shock state upon initial admission, resulted in a decreased rate of OM with a similar mortality rate [24]. However, it must be emphasized that in these management options, there should be rapid availability of an operating team if the patients’ conditions deteriorate. On the other hand, in trauma centers where IR facilities were not promptly available, only 6% of the HD unstable patients underwent the TAE procedure [25].

Hemorrhage control is time-critical, emphasized by the data demonstrating that delays in operative intervention in patients with significant abdominal injuries caused a 1% higher mortality risk for every 3 min of delay in reaching the operating room [26]. Damage control surgery in liver trauma is based on the surgical dogma “Push, Pack and Pringle”, which summarizes the main surgical maneuvers that surgeons must perform to limit bleeding. Most venous bleeding could be controlled by a liver packing procedure; however, arterial injury could continue to produce bleeding, and TAE, associated with packing, may rule out hemorrhage control [27]. For this reason, the choice of which patients could benefit from an immediate operative management versus angiographic study is critical, especially with these partial responder unstable patients where time is everything; the development of hybrid operating rooms, which allow surgeons to perform multiple bleeding control procedures in the same location, eliminating the need to move patients back and forth between rows, showed potential reductions in mortality and procedure time. The RAPTOR study demonstrated a significant reduction in treatment time, with about 18% of patients requiring an emergent percutaneous procedural intervention added to open surgery and showing a clear benefit for survival (42% RAPTOR era vs. 22% pre-RAPTOR era) in a hybrid suite. However, this study postulated that the cost associated with a hybrid suite, where an advanced angiography system is available, remains prohibitive for many centers [28,29]. Another cheaper solution could be the use of a mobile C-arm in an operating room equipped with a carbon-fiber fluoroscopic table [11].

The resuscitative endovascular balloon occlusion of the aorta (REBOA) could be another useful strategy in HD unstable patients that require direct OM; however, the evidence base on its use in liver trauma patients is weak and there is no clear indication on the time and zone of balloon inflation. Its use in HD unstable patients with multiple severe torso trauma refractory undergoing initial infusion therapy has been reported to improve prognosis [14].

To the best of our knowledge, this is one of the first systematic reviews that summarizes patient outcomes regarding TAE in HD unstable/transient responder liver trauma patients. Nevertheless, there are some limitations to this study that should be noted. First, the total sample size was low, which lowered the validity of the present results and indicates that large-scale studies on this topic, especially prospective studies, are needed. Second, the majority of the included studies are retrospective or observational studies. Therefore, high-quality trials to explore the efficacy and safety of angioembolization in this setting are needed. Finally, we did not study the selection criteria of patients for angioembolization in this setting.

## 5. Conclusions

IR is a fundament of trauma patients’ care and management; one of the greatest limitations of endovascular treatment is represented by being classified as a NOM. TAE should be compared to a surgical operation, with its well-known risks and complications, and it should be no longer counted as NOM but rather as OM or differentiated by creating a new section management known as endovascular treatment. To date, the role of IR in the management and treatment of patients with severe liver trauma has been well known; our review demonstrated a very good outcome in HD unstable/transient responder patients treated with TAE. However, there still remains much debate about the definition of unstable patients and transient responder patients to resuscitative treatment because these are borderline situations in which it is very difficult to identify clear parameters, both clinical and biochemical. Using TAE in unstable/transient responder liver trauma patients is feasible, but more prospective studies, even better if they are multicentric, are needed to standardize the treatment and also to clear the fog that exists regarding the definition of the HD status of patients.

## Figures and Tables

**Table 1:** General characteristics of the studies included in the review.

Authors	Year	Study Type	Total Patients	M/F Ratio	Mean Age	Total Primary Embolization in Unstable Patients	% Grade Trauma (AAST)
Tamura S et al. [9]	2021	RT	92	3.7/1	29.5	59	Grade III (58%) Grade IV (34%) Grade V (8%)
Aoki M et al. [10]	2021	RT	224	1.5/1	55	57	Grade III (40%) Grade IV (18%) Grade V (5%)
Alnumay A et al. [11]	2021	RT	49	4/1	44	5	Not available
Inukai K et al. [12]	2018	RT	23	1/1	32.3	10	Grade IV (80%) Grade V (20%)
Otsuka et al. [13]	2017	OB	16	3/1	46	5	Grade III (40%) Grade IV (20%) Grade V (40%)
Ogura et al. [14]	2014	RT	7	3/1	63.5	3	Grade II (33%) Grade IV (34%) Grade V (33%)
Mitsusada M et al. [15]	2013	RT	29	2.2/1	38.5	8	Not available
Di Saverio S et al. [16]	2012	RT	34	1.5/1	42	10	Not available
Misselbeck TS et al. [17]	2009	RT	21	N/A	N/A	11	Not available
Monnin et al. [18]	2007	RT	12	N/A	35	3	Grade IV (100%)

**Table 2:** Different definitions of hemodynamic stability and instability.

Authors	Year	Definition of Stable Patient	Definition of Unstable Patient
Tamura S et al. [9]	2021	All trauma patients responding to initial standard infusion therapy (crystalloid, albumin and blood transfusion)	SI > 1, despite initial infusion therapy
Aoki M et al. [10]	2021	sBP ≥ 90 mmHg upon hospital arrival	sBP < 90 mmHg upon hospital arrival and received blood transfusion within the first 24 h after arrival
Alnumay A et al. [11]	2021	Not available	Not available
Inukai K et al. [12]	2018	sBP ≥ 90 mmHg after initial fluid treatment	sBP ≥ 90 mmHg for even a second and therefore required rapid fluid infusion or blood transfusion
Otsuka et al. [13]	2017	sBP ≥ 90 mmHg after initial fluid treatment	sBP < 90 mmHg without improvement following primary resuscitation
Ogura et al. [14]	2014	Not available	sBP maintained at 70 mm Hg or greater during deflation of the balloon (REBOA)
Mitsusada M et al. [15]	2013	Not available	sBP ≥ 80 mmHg after resuscitative therapy
Di Saverio S et al. [16]	2012	Not available	Not available
Misselbeck TS et al. [17]	2009	At admission, sBP was ≥ 90 mm Hg and intravenous fluid requirements did not exceed 2 L.	Not available
Monnin et al. [18]	2007	Patients who were hemodynamically stable or stabilized by low or moderate resuscitation.	Patients with hemorrhagic shock improved or stabilized after resuscitative treatment.

**Table 3:** Rate and type of mortality and morbidity reported in individual studies.

Authors	Year	Total Number of Embolized Patients *N*	Mortality *N* (%)	AE Failure *N* (%)	Post-AE Complications *N* (%)
Tamura S et al. [9]	2021	59	3 (3.2)	2 (2.1)	29 (31.5)
Aoki M et al. [10]	2021	57	7 (12)	Not available	9 (15.7)
Alnumay A et al. [11]	2021	5	Not available	Not available	Not available
Inukai K et al. [12]	2018	10	1 (10)	0	5 (50)
Otsuka et al. [13]	2017	5	Not available	1 (20)	4 (80)
Ogura et al. [14]	2014	3	1 (33)	Not available	Not available
Mitsusada M et al. [15]	2013	8	0	0	3 (37.5)
Di Saverio S et al. [16]	2012	10	Not available	Not available	Not available
Misselbeck TS et al. [17]	2009	11	4 (36)	1 (9)	9 (81)
Monnin et al. [18]	2007	3	N/A	N/A	N/A
TOTAL		171	16 (9.35)	4 (2.33)	59 (34.5)

**Table 4:** Rate and type of complications reported in individual studies.

Authors	Year	Total Number of Embolized Patients *N*	Liver Abscess/Biloma *N* (%)	Bile Leakage *N* (%)	Gallbladder Necrosis/Cholecystitis *N* (%)	Peritonitis *N* (%)	Abdominal Compartment Syndrome *N* (%)
Tamura S et al. [9]	2021	59	13 (14.1)	0	1 (1.08)	0	0
Aoki M et al. [10]	2021	57	3 (5.26)	0	1 (1.7)	3 (5.2)	Not available
Alnumay A et al. [11]	2021	5	Not available	Not available	Not available	Not available	Not available
Inukai K et al. [12]	2018	10	2 (20)	1 (10)	0	0	2 (20)
Otsuka et al. [13]	2017	5	0	0	1 (20)	2 (40)	0
Ogura et al. [14]	2014	3	Not available	Not available	Not available	Not available	Not available
Mitsusada M et al. [15]	2013	8	0	3 (37.5)	0	0	0
Di Saverio S et al. [16]	2012	10	Not available	Not available	Not available	Not available	Not available
Misselbeck TS et al. [17]	2009	11	4 (36)	1 (9)	6 (66)	6 (54)	0
Monnin et al. [18]	2007	3	Not available	Not available	Not available	Not available	Not available
TOTAL		171	22 (12.8)	5 (3)	9 (5.3)	11 (6.4)	2 (1.16)

## Data Availability

No new data were created or analyzed in this study. Data sharing is not applicable to this article.
